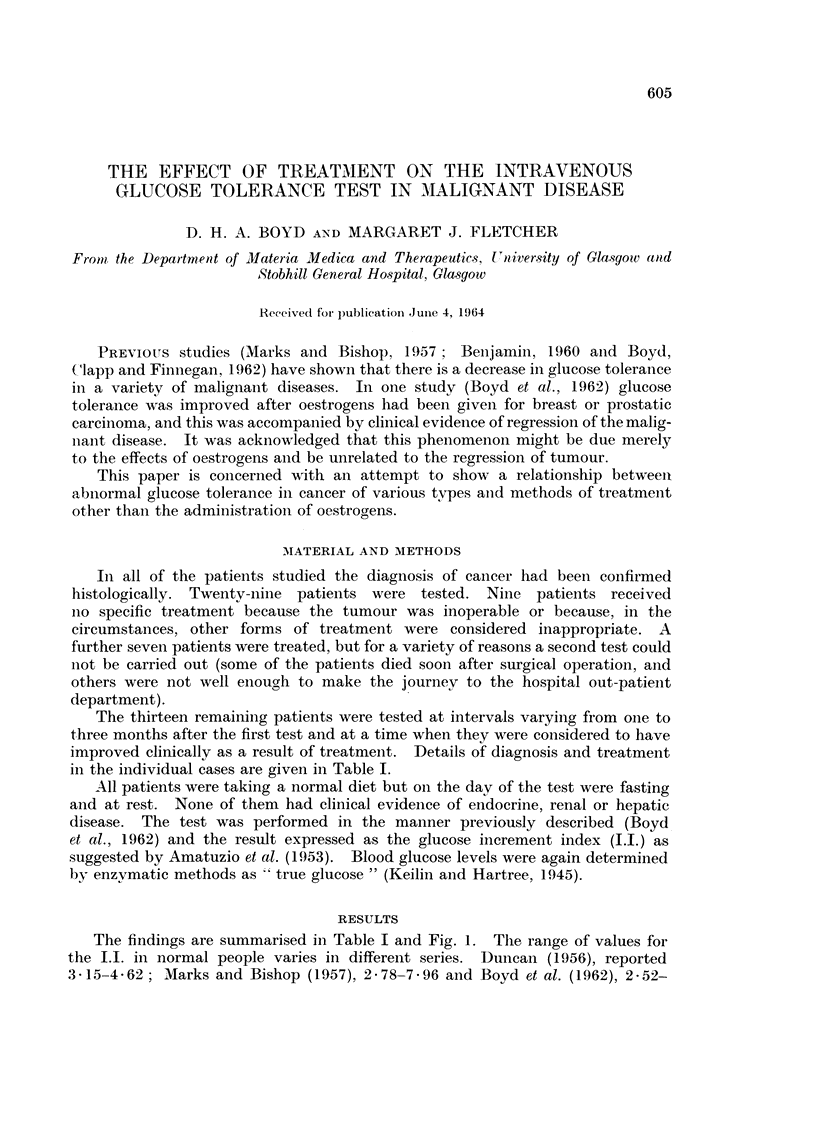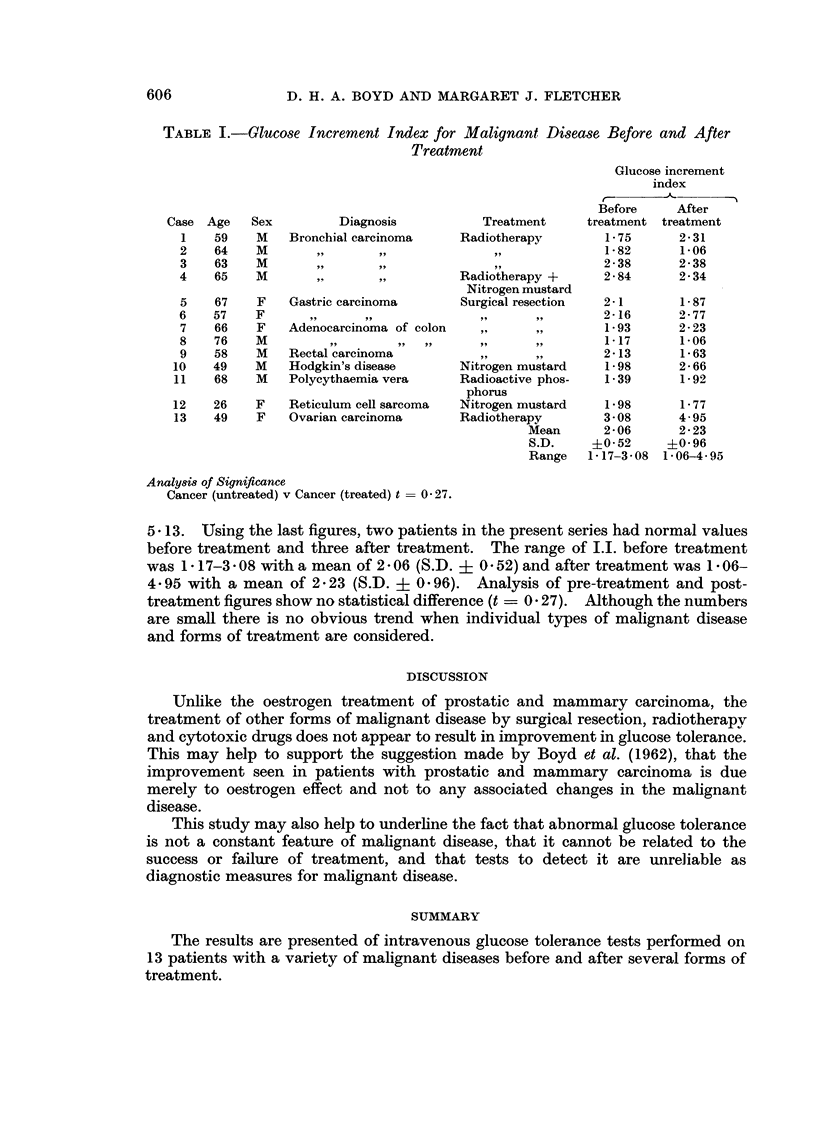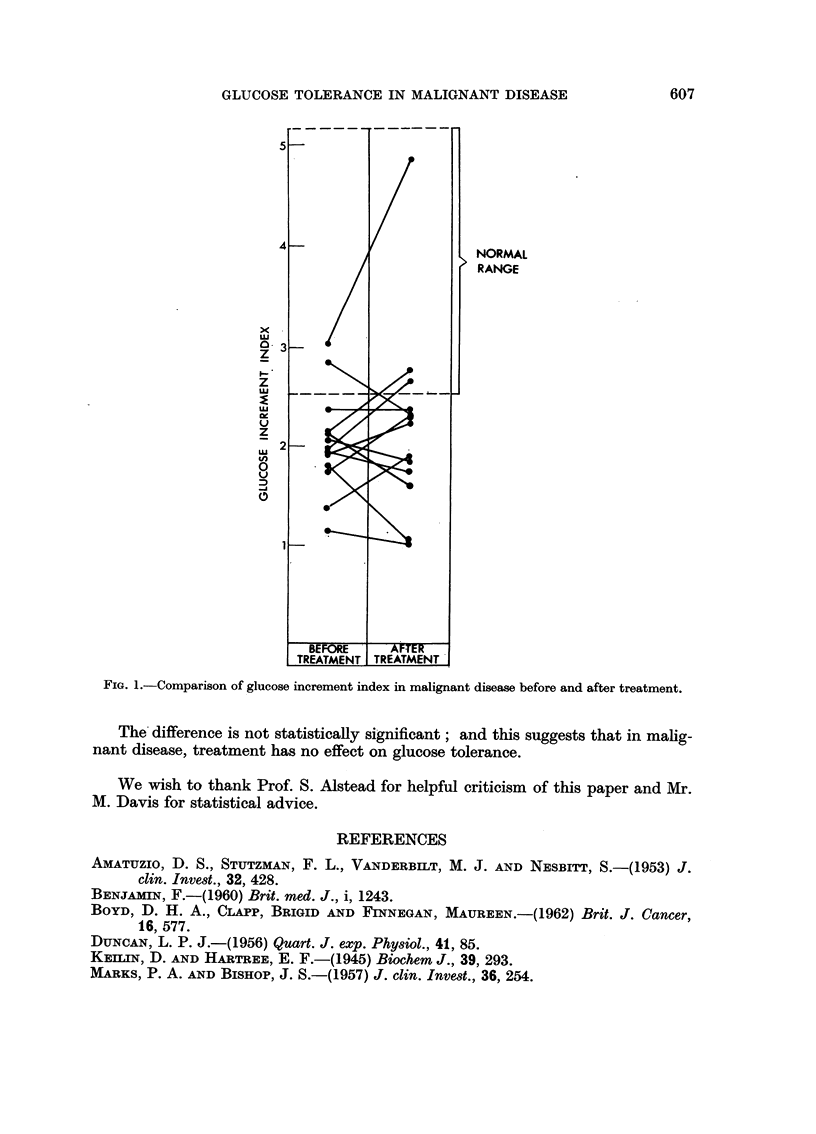# The Effect of Treatment on the Intravenous Glucose Tolerance Test in Malignant Disease

**DOI:** 10.1038/bjc.1964.69

**Published:** 1964-09

**Authors:** D. H. A. Boyd, Margaret J. Fletcher


### THE EFFECT OF TREATMENT ON THE INTRAVENOUS GLUCOSE TOLERANCE TEST IN MALIGNANT DISEASE

D. H. A. BOYD AND MARGARET J. FLETCHER

*From the Department of Materia Medica and Therapeutics, University of Glasgow and Stobhill General Hospital, Glasgow*

Received for publication June 4, 1964

PREVIOUS studies (Marks and Bishop, 1957; Benjamin, 1960 and Boyd, Clapp and Finnegan, 1962) have shown that there is a decrease in glucose tolerance in a variety of malignant diseases. In one study (Boyd *et al.*, 1962) glucose tolerance was improved after oestrogens had been given for breast or prostatic carcinoma, and this was accompanied by clinical evidence of regression of the malignant disease. It was acknowledged that this phenomenon might be due merely to the effects of oestrogens and be unrelated to the regression of tumour.

This paper is concerned with an attempt to show a relationship between abnormal glucose tolerance in cancer of various types and methods of treatment other than the administration of oestrogens.

### MATERIAL AND METHODS

In all of the patients studied the diagnosis of cancer had been confirmed histologically. Twenty-nine patients were tested. Nine patients received no specific treatment because the tumour was inoperable or because, in the circumstances, other forms of treatment were considered inappropriate. A further seven patients were treated, but for a variety of reasons a second test could not be carried out (some of the patients died soon after surgical operation, and others were not well enough to make the journey to the hospital out-patient department).

The thirteen remaining patients were tested at intervals varying from one to three months after the first test and at a time when they were considered to have improved clinically as a result of treatment. Details of diagnosis and treatment in the individual cases are given in Table I.

All patients were taking a normal diet but on the day of the test were fasting and at rest. None of them had clinical evidence of endocrine, renal or hepatic disease. The test was performed in the manner previously described (Boyd *et al.*, 1962) and the result expressed as the glucose increment index (I.I.) as suggested by Amatuzio *et al.* (1953). Blood glucose levels were again determined by enzymatic methods as " true glucose " (Keilin and Hartree, 1945).

### RESULTS

The findings are summarised in Table I and Fig. 1. The range of values for the I.I. in normal people varies in different series. Duncan (1956), reported 3·15–4·62; Marks and Bishop (1957), 2·78–7·96 and Boyd *et al.* (1962), 2·52–

TABLE I.—*Glucose Increment Index for Malignant Disease Before and After Treatment*

| Case | Age | Sex | Diagnosis | Treatment | Glucose increment index | |
|---|---|---|---|---|---|---|
| | | | | | Before treatment | After treatment |
| 1 | 59 | M | Bronchial carcinoma | Radiotherapy | 1·75 | 2·31 |
| 2 | 64 | M | ,, ,, | ,, | 1·82 | 1·06 |
| 3 | 63 | M | ,, ,, | ,, | 2·38 | 2·38 |
| 4 | 65 | M | ,, ,, | Radiotherapy + Nitrogen mustard | 2·84 | 2·34 |
| 5 | 67 | F | Gastric carcinoma | Surgical resection | 2·1 | 1·87 |
| 6 | 57 | F | ,, ,, | ,, ,, | 2·16 | 2·77 |
| 7 | 66 | F | Adenocarcinoma of colon | ,, ,, | 1·93 | 2·23 |
| 8 | 76 | M | ,, ,, ,, | ,, ,, | 1·17 | 1·06 |
| 9 | 58 | M | Rectal carcinoma | ,, ,, | 2·13 | 1·63 |
| 10 | 49 | M | Hodgkin's disease | Nitrogen mustard | 1·98 | 2·66 |
| 11 | 68 | M | Polycythaemia vera | Radioactive phosphorus | 1·39 | 1·92 |
| 12 | 26 | F | Reticulum cell sarcoma | Nitrogen mustard | 1·98 | 1·77 |
| 13 | 49 | F | Ovarian carcinoma | Radiotherapy | 3·08 | 4·95 |
| | | | | Mean | 2·06 | 2·23 |
| | | | | S.D. | ±0·52 | ±0·96 |
| | | | | Range | 1·17–3·08 | 1·06–4·95 |

*Analysis of Significance*
Cancer (untreated) v Cancer (treated) $t = 0 \cdot 27$.

5·13. Using the last figures, two patients in the present series had normal values before treatment and three after treatment. The range of I.I. before treatment was 1·17–3·08 with a mean of 2·06 (S.D. ± 0·52) and after treatment was 1·06–4·95 with a mean of 2·23 (S.D. ± 0·96). Analysis of pre-treatment and post-treatment figures show no statistical difference ($t = 0 \cdot 27$). Although the numbers are small there is no obvious trend when individual types of malignant disease and forms of treatment are considered.

### DISCUSSION

Unlike the oestrogen treatment of prostatic and mammary carcinoma, the treatment of other forms of malignant disease by surgical resection, radiotherapy and cytotoxic drugs does not appear to result in improvement in glucose tolerance. This may help to support the suggestion made by Boyd *et al.* (1962), that the improvement seen in patients with prostatic and mammary carcinoma is due merely to oestrogen effect and not to any associated changes in the malignant disease.

This study may also help to underline the fact that abnormal glucose tolerance is not a constant feature of malignant disease, that it cannot be related to the success or failure of treatment, and that tests to detect it are unreliable as diagnostic measures for malignant disease.

### SUMMARY

The results are presented of intravenous glucose tolerance tests performed on 13 patients with a variety of malignant diseases before and after several forms of treatment.

FIG. 1.—Comparison of glucose increment index in malignant disease before and after treatment.

The difference is not statistically significant; and this suggests that in malignant disease, treatment has no effect on glucose tolerance.

We wish to thank Prof. S. Alstead for helpful criticism of this paper and Mr. M. Davis for statistical advice.

### REFERENCES

AMATUZIO, D. S., STUTZMAN, F. L., VANDERBILT, M. J. AND NESBITT, S.—(1953) *J. clin. Invest.*, **32**, 428.

BENJAMIN, F.—(1960) *Brit. med. J.*, i, 1243.

BOYD, D. H. A., CLAPP, BRIGID AND FINNEGAN, MAUREEN.—(1962) *Brit. J. Cancer*, **16**, 577.

DUNCAN, L. P. J.—(1956) *Quart. J. exp. Physiol.*, **41**, 85.

KEILIN, D. AND HARTREE, E. F.—(1945) *Biochem J.*, **39**, 293.

MARKS, P. A. AND BISHOP, J. S.—(1957) *J. clin. Invest.*, **36**, 254.